# COGNITION IN CONTEXT: INVESTIGATING THE ROLE OF BUILT, SOCIAL, AND NATURAL ENVIRONMENTS IN COGNITIVE AGING

**DOI:** 10.1093/geroni/igac059.024

**Published:** 2022-12-20

**Authors:** Jessica Finlay, Philippa Clarke, Lilah Besser

## Abstract

While a growing body of evidence points to potentially modifiable individual risk factors for Alzheimer’s Disease and Related Dementias (ADRD), the contexts in which people develop and navigate cognitive decline are largely overlooked. Geographic variation in ADRD rates suggest that environmental risk and protective factors may be important in cognitive aging and dementia caregiving. Community hazards are often heavily concentrated in underserved and underrepresented neighborhoods. This symposium aims to identify specific built, social, and natural environmental features associated with cognitive aging outcomes. The papers provide much-needed evidence on the role of neighborhoods and community networks for cognitive health and well-being among diverse older adults. First, Godina finds significant associations between neighborhood greenspace and microstructural indicators of brain health. Second, Westrick investigates the role of neighborhood disadvantage on long-term memory aging of older adults with and without a cancer diagnosis in later-life. Third, Finlay presents a new concept of Cognability to demonstrate which constellation of positive and negative neighborhood features may contribute most to healthy cognitive aging. Fourth, Nkimbeng identifies community networks and resources needed to inform dementia education and support care among African immigrants. Fifth, expert discussant Besser will share how these findings may inform upstream health promotion and reduce ADRD risk. She will discuss critical future research directions and methods to investigate environments of cognition. The symposium advances research assessing contexts of aging, and may inform public health and policy efforts to ameliorate community barriers and create more equitable opportunities to promote healthy aging in place.

